# Profile of cyclists with head injury admitted to a London Major Trauma Centre

**DOI:** 10.1186/1757-7241-23-S2-A12

**Published:** 2015-09-11

**Authors:** Anna E Forbes, John M Schutzer-Weissmann, Matthew Wordsworth, Mark H Wilson

**Affiliations:** 1 Intensive Care Unit and Major Trauma Ward, St Mary's Hospital, Imperial College Healthcare NHS Trust, London, UK

## Background

As the number of cyclists in Britain continues to grow [1], interest in cycle safety has increased. The aim of this study was to identify patient characteristics, mechanisms of injury and injury patterns in cyclists with head injury admitted to a London Major Trauma Centre with a view to identifying factors which could increase cycle safety.

## Methods

Cyclists with any head injury requiring admission to hospital between January 1^st^ 2011 and December 31^st^ 2013 were identified from Intensive Care admissions, Emergency Department records, the Trauma Audit and Research Network and patient lists for the Trauma Ward.

After identification of the patient group, data was collected from emergency department and pre-hospital documentation, imaging, toxicology and Intensive Care documentation where relevant.

## Results

93 patients were identified with an average age of 37. 89% were male. 54% were not wearing helmets, 20% were wearing helmets and helmet use was not recorded for 26%. The most common mechanisms of injury were cyclists vs car (41%) and falls (38%).

There was no pattern of laterality in terms of intracranial or extracranial injuries. In those with intracranial injury 53% sustained contusions, 49% Subarachnoid haemorrhage, 38% subdural haematoma (SDH) and 23% extradural haematoma (EDH). Where helmet use was recorded no patients wearing a helmet sustained an EDH and only 1 had a SDH.

25% of patients had a significant alcohol history or positive alcohol level where 27% had negative alcohol levels, the remainder had no levels sent.

62% of those not wearing a helmet suffered intracranial injuries compared to 32% of those who were.

## Conclusions

Although numbers in this study are small these results suggest that helmet use is protective against intracranial injury.

Additionally 25% of those admitted to hospital with head injury were intoxicated, suggesting that this a risk factor in serious cycling accidents.
